# Age-dependent role of steroids in the regulation of growth of the hen follicular wall

**DOI:** 10.1186/1477-7827-8-15

**Published:** 2010-02-15

**Authors:** Irina Y Lebedeva, Vladimir A Lebedev, Roland Grossmann, Nahid Parvizi

**Affiliations:** 1 Department of Functional Genomics and Bioregulation, Institute of Animal Genetics, FLI, Mariensee, 31535 Neustadt, Germany; 2 Center of Biotechnology and Molecular Diagnostics, Russian Research Institute of Animal Breeding, Podolsk, 142132 Russia; 3 Department of Genetics and Biotechnology, Research Institute for Farm Animal Genetics and Breeding, Pushkin, St Petersburg, 196625 Russia

## Abstract

**Background:**

The ovaries are the primary targets of senescence effects in mammalian and avian species. In the present study, relationships between reproductive aging, sex steroids and the growth pattern of the pre-ovulatory follicle wall were investigated using young hens with long clutch (YLC), old hens with long clutch (OLC), old hens with short clutch (OSC), and old hens with interrupted long clutch (OILC).

**Methods:**

Experiment 1: Hens were sacrificed 1.5 and 14.5 h after ovulation. Experiment 2: YLC and OILC hens were sacrificed 3.5 h after treatments with LH and/or aminoglutethimide (AG), an inhibitor of steroid synthesis. Volumes of pre-ovulatory follicles (F1-F5) and plasma concentrations of ovarian steroids were determined. Experiment 3: Granulosa and theca cells from F3 follicles of OSC and/or YLC hens were exposed in vitro to estradiol-17beta (E_2_), testosterone (T) and LH and the proliferative activity of the cells was examined using CellTiter 96 Aqueous One Solution Assay.

**Results:**

In YLC and OLC groups, the total volume of F1-F5 follicles rose between 1.5 and 14.5 h after ovulation (P < 0.01), negatively correlating with the plasma level of E_2 _(P < 0.01). There was no growth of pre-ovulatory follicles in the middle of the ovulatory cycle in the OSC group, with a positive correlation being present between E_2 _and the follicular volume (P < 0.05). In young hens, AG caused a rise in the total follicular volume. This rise was associated with a fall in E_2 _(r = -0.54, P < 0.05). E_2 _enhanced proliferation of granulosa cells from YLC and OSC groups. The proliferative activity of granulosa and theca cells of YLC hens depended on the interaction between T and LH (P < 0.01).

**Conclusions:**

These data indicate for the first time that the growth pattern of pre-ovulatory follicles during the ovulatory cycle changes in the course of reproductive aging. E_2 _seems to play a dual role in this adjustment; it stimulates the growth of the follicular wall in reproductive aged hens, whereas it may inhibit this process in young birds. T and LH are apparently involved in the growth regulation during the pre-ovulatory surge in young hens.

## Background

The reproductive performance in females is known to deteriorate with age. The ovaries and particularly the follicles are the primary targets of senescence effects [1]. In women and laboratory rodents, the initial decline in the ovarian function associated with the depletion of the follicular reserve occurs in the midlife followed by the gradual transition to the menopause or anestrous state [2-6]. Age-dependent alterations in the ovarian activity are closely connected with endocrine changes, primarily with variations in circulating levels of gonadotropins and sex steroids [7,8]. The currently available evidence suggests that dynamics of the follicular growth changes in mammals of advanced reproductive age. As this takes place, preovulatory follicles grow more slowly and attain a smaller diameter at ovulation in older women than they do in younger ones [9,10]. In contrast, pre-ovulatory follicles are larger and the proliferative activity of granulosa cells is higher in aged than in young rats [3,11]. Both of these phenomena can be, at least in part, due to age-related alterations in the production of ovarian steroids and/or in the follicular sensitivity to these steroids [7,8].

As in the case of mammals, the reproductive performance decreases gradually in hens during aging, with a decrease in the length of clutch and an increase in the interval between ovulations. This already occurs at the end of the first year of laying [12,13]. Another sign of impaired reproductive function in aged hens is the enhanced frequency of missing eggs/anovulatory cycles within clutch [13]. The growth pattern of hen pre-ovulatory follicles is also affected by senescence. The length of the rapid growth period is prolonged and the size of the biggest pre-ovulatory follicle is enlarged after 60-70 weeks of age [14-16]. However, no obvious differences in circulating levels of ovarian steroids have been found between young and aged layers [15,16]. Thus, it has been suggested that changes in the follicular growth in older hens may result from more subtle changes in levels of sex steroids, such as time or amplitude of the pre-ovulatory surge of hormones [15].

We have shown previously that the ovulatory cycle-related alterations in the thecal weight and membrane protein content occur in pre-ovulatory follicles of young laying hens [17]. Concurrently, the thecal growth is transitorily ceased, whereas the mean membrane protein in the theca increases shortly before ovulation of the largest follicle. This suggests that the pre-ovulatory rise in LH and/or ovarian steroid hormones is probably involved in the short-time termination of the growth and induction of differentiation of the theca in pre-ovulatory follicles as they pass from one category to the next. The present study was conducted to test a hypothesis that the growth of the whole follicular wall consisting of both theca and granulosa layers is adjusted to the hen ovulatory cycle and the pattern of this adjustment changes during reproductive aging. Furthermore, we examined which sex steroids may be involved in the regulation of the tissue growth of pre-ovulatory follicles in relation to the ovulatory cycle and reproductive aging.

## Methods

### Animals

Lohmann Selected Leghorn (LSL, Lohmann, Cuxhaven, Germany) hens (Gallus domesticus) were kept in individual cages under a lighting regimen of 12 h light: 12 h dark with free access to food and water. Egg laying was recorded daily for each hen and the following groups of birds were selected for experiments: (1) young hens (29-31 week-old) with long clutch (YLC, >10 eggs per clutch; interval between ovulations, io = 24.0 ± 0.1 h), (2) old hens (62-82 week-old) with long clutch (OLC, >10 eggs per clutch; io = 24.1 ± 0.1 h), (3) old hens with short clutch (OSC, 3-6 eggs per clutch; io = 25.6 ± 0.2 h), and (4) old hens with interrupted long clutch (OILC, >10 eggs per clutch; io = 24.4 ± 0.2 h). Hens of the OILC group were determined as those laying 3-6 eggs between anovulatory cycles within long clutch and lacking a rise in the interval between ovulations. Missing eggs within long clutch were visible signs of anovulatory cycles [13], which were confirmed by the presence of large regressing non-ovulated follicles in the ovaries after hen slaughtering. In contrast, hens of the OSC group had an increased interval between ovulations and lacked regressing non-ovulated follicles. Time of oviposition was monitored by visual inspection in 20-min intervals from the beginning of the clutch preceding the clutch under study. Time of ovulation was estimated on the basis that it occurs approximately 30 min after oviposition [18]. The interval between ovulations was determined as the time lapse between successive ovipositions within clutch (YLC, OLC and OSC) or within uninterrupted egg sequences inside clutch (OILC). These intervals were used to calculate time before expected ovulation of pre-ovulatory follicles of different categories. Hens were sacrificed by cervical dislocation in the middle of their clutch (YLC, OLC and OSC) or uninterrupted sequence of clutch (OILC). Birds were used in accordance with procedures approved by the regional Animal ethics Committee (approval no. 509C 42502 00/394).

### Experimental design

#### Experiment 1

Hens were sacrificed at 1.5 h (stage I; YLC and OLC: n = 6, OSC: n = 10) and 14.5 h (stage II; YLC, OLC, and OSC: n = 6) after ovulation. Ovulation was confirmed by the presence of an egg yolk in the oviduct. We had previously found significant differences in the thecal weight between these two time points of the ovulatory cycle in young hens [17].

#### Experiment 2

Hens were (YLC, OILC) treated with ovine luteinizing hormone (oLH; NIAMDD-oLH-23) and/or aminoglutethimide (AG; Sigma Chemical Co, St. Louis, MO), an inhibitor of steroid synthesis which interrupts the cholesterol side-chain cleavage by the cytochrome P-450 [19]. Birds of the YLC group weighing 1.56 ± 0.02 kg were randomly divided into four treatment groups: Control (n = 9), AG (n = 9), LH (n = 7), LH+AG (n = 8). The OILC hens were randomly divided into two treatment groups: Control (n = 7) and AG (n = 7). The mean weight of these hens was 1.65 ± 0.03 kg. The hens were subcutaneously injected with saline or 100 mg AG at 9.5 and 11.5 h after ovulation. This dose of AG sufficed to decrease circulating steroid levels in hens without causing a physiological stress [20]. Immediately after the second treatment with AG, the hens were intravenously injected with saline or 100 μg oLH. The hens were sacrificed at 3.5 h after the treatment with AG or oLH, corresponding to 15 h after ovulation. The schedule of the treatments is given in Table 1.

**Table 1:** Schedule of injections

Treatment group	1st injection: 100 mg AG^**1**^ (9.5 h after ovulation)	2nd injection: 100 mg AG ^**1**^ (11.5 h after ovulation)	Injection: 100 μg oLH ^**2**^ (immediately after the 2nd injection of AG)	Slaughtering (3.5 h after the injection of oLH)
Control	s	s	s	+
AG	+	+	s	+
LH	s	s	+	+
LH + AG	+	+	+	+

1 = subcutaneous injection; 2 = intravenous injection; s = saline.

#### Experiment 3

Hens (YLC: n = 11 and OSC: n = 5) were sacrificed at 7 h after ovulation. This time point was chosen (a) to permit restoring of the sensitivity of follicular cells to sex steroids and LH after the pre-ovulatory hormone surge, and (b) because, during this period of the ovulatory cycle the follicular theca grows significantly [17].

Based on the results of experiment 1 and as a consequence of limited number of old hens having regular short clutches (OSC) or having regular anovulatory cycles within long clutches (OILC), not all groups of the animals were used in all three experiments.

### Blood sampling, follicular size measurement and tissue collection

Immediately after slaughtering, blood samples were collected from the heart in heparin-coated tubes. Plasma samples were frozen and stored at -20°C for estradiol-17β, testosterone and progesterone measurements.

Ovaries were removed and rinsed in saline. The first (F1), second (F2), third (F3), fourth (F4) and fifth (F5) largest yellow follicles were detached from the ovaries. The diameters of the pre-ovulatory follicles were measured along and across the stigma. The growth of the follicular wall was examined by comparison of the size of follicles of different categories at distinct stages of the ovulatory cycle. The follicular size has been shown to be a better criterion of the follicular maturity than the weight of yolk-free mass [21]. The size of the follicular wall was determined as a surface area of the follicle and calculated, assuming a follicle shape as an ellipsoid, on the basis of the following equation: S = 2πD_2 _× [(2(D_1_/2)^p ^+ (D_2_/2)^p^)/3]^1/p^, where D_1 _was the diameter along the stigma, D_2 _was the diameter across the stigma and p = 1.6075. Simultaneously, the volume of the respective follicles was calculated by application of the equation: V = π × D_1 _× D_2 _^2^/6. Preliminary experiments revealed that the correlation coefficient between the follicular volume and surface area calculated using the above equations was 0.997 (*P *< 0.001, n = 60). Therefore, the growth of the follicular wall was further estimated based on the differences in the follicular volume.

The third largest yellow follicles (F3) of hens sacrificed in Experiment 3 were immediately placed in warm (37°C) sterile saline. Theca and granulosa layers were separated as described by Gilbert et al. [22] and rinsed several times in warm Dulbecco's PBS containing 50 μg/ml gentamicin sulfates to remove any adhering yolk.

### Cell isolation

Follicular cells were isolated as described by Tilly and Johnson [23] and Roberts et al. [24] with some modifications. Theca and granulosa layers were placed in sterile glass vials containing 2 ml of warmed (39°C) 0.3% collagenase (type 2, w/v; CellSystems Biotechnologie Vertrieb GmbH, Germany) solution in a basic medium and minced with fine scissors. DMEM (Sigma) supplemented with 1 g/l glucose (Sigma), 2.5 mg/ml BSA (Sigma), 25 mM HEPES (Carl Roth GmbH and Co, Karlsruhe, Germany), and 10 ml/l antibiotic antimycotic solution (Sigma) was used as the basic medium.

Granulosa tissues were re-suspended with a Pasteur pipette and digested for 5 min at 39°C. After incubation, the cells were dispersed with the pipette and pelleted by centrifugation at 200 × g for 15 min (20°C). The supernatant was discarded and the cells were re-suspended in 3 ml of the fresh basic medium without collagenase and centrifuged. The washing procedure was repeated twice. After the last centrifugation, the cells were dispersed in the following culture medium: DMEM supplemented with 1 g/l glucose, 25 mM HEPES, 10 ml/l antibiotic antimycotic solution, and 20 ml/l serum replacement 1 (Sigma) containing BSA, bovine transferrin, and bovine insulin. Granulosa cells proximal and distal to the germinal disk were components of the cellular suspension used, because both cell types make a contribution to the growth of the granulosa layer [25].

Theca tissues were digested for 1 h at 39°C in a shaking water bath. The upper layer (700-800 μl) of the suspension was transferred to a test tube, diluted with 2 ml of the fresh basic medium (without collagenase), and centrifuged at 300 × g for 15 min (20°C). The pellet was washed with 3 ml of the medium and centrifuged again. The cells were re-suspended in 2 ml of the basic medium followed by the addition of 2 ml of Percoll (Sigma) to produce a 50% Percoll solution (v/v). This mixture was centrifuged at 400 × g for 25 min (20°C) to separate theca cells from red blood cells and clumps of the tissue. The top layer containing thecal cells was carefully removed and pelleted by centrifugation at 300 × g for 15 min. The cells were washed three times by centrifugation with the basic medium. After the last centrifugation, the cells were re-suspended in the culture medium. The final cellular suspension used for culture was a mixture of different theca cell types, since they all contribute to the growth of the theca tissue [26].

The number and viability of follicular cells were determined with a hemocytometer using the trypan blue exclusion technique. Viability of granulosa and theca cells was always greater than 90 and 85%, respectively.

### Cell culture

Granulosa and theca cells from one follicle of YLC (n = 5) and OSC (n = 5) hens were seeded simultaneously in two 96-well plastic plates at a final concentration of 40,000 cells per well and cultured for 0 and 40 h under a humidified atmosphere of 5% CO_2 _in air at 39°C in 100 μl of the above culture medium containing 0 or 1 ng/ml estradiol-17β (Sigma). In addition, the cells of YLC (n = 6) hens were cultured in the absence (Control) or presence of 10 ng/ml testosterone (Sigma), 10 ng/ml oLH or both hormones simultaneously. Thereafter, the cells were lysed with CellTiter 96 Aqueous One Solution for cell number measurement. Five to six independent culture sets were conducted for each group of hens (YLC, OSC), each containing three replicates per treatment. Granulosa and theca cells from F3 follicle of one hen were used for one culture set.

### Measurement of proliferative activity of follicular cells

Determination of the viable cell number was carried out using CellTiter 96 Aqueous One Solution Cell Proliferation Assay (Promega, Madison, WI) as described by Yao and Bahr [25] with some modifications. After 0 or 40 h of culture, 20 μl of One Solution Reagent were added to each well followed by 3 h of incubation at 39°C. Preliminary experiments revealed that this incubation time suffices for the complete cellular lysis and that the number of viable cells is directly related to the absorbance of the medium, when read with a spectrophotometer at 450 nm. For each treatment, the absorbance in wells containing the medium without cells was subtracted from the absorbance in wells containing the medium with cells. The relative number of viable cells after 40 h of culture was expressed in percent of the number of the cells prior to culture (0 h).

### Hormone determination

Plasma concentrations of estradiol-17β (E_2_), progesterone (P_4_), and testosterone (T) were measured by enzyme immunoassay [27, see also erratum of this paper]. These assays have been validated for measurements of E_2_, P_4 _and T in the blood plasma of chicken. The curves using aliquots of different dilutions of the chicken plasma were parallel to the standard curves. The recovery of 0.25, 5 and 10 ng/ml E_2 _added to the plasma of hens was 88.6%, 106% and 101% respectively. The recovery of 1.25, 5 and 20 ng/ml P_4 _added to the plasma of hens was 93%, 111%, and 103% and the recovery of 0.6, 2.5 and 5 ng/ml T added to the hen plasma was 114%, 103% and 89.4% respectively. The sensitivity of the assays was 50 pg/ml for E_2_, 125 pg/ml for P_4 _and 100 pg/ml for T. The intra-assay coefficients of variation for E_2_, P_4 _and T-assay were 9%, 12.2% and 6.8% respectively. All samples for measurements of one hormone were analyzed in the same assay.

### Statistical analysis

Results are expressed as means ± SEM. Data on the volumes of follicles with various degrees of maturation in hens at different stages of the ovulatory cycle were analyzed by two-way repeated-measures ANOVA using SAS software. The independent variables were the stage of the ovulatory cycle and follicular category (repeated factor), with the hen nesting within the follicular category (an error term). Plasma levels of E_2_, T, and P_4 _and the total follicular volume in hens of the YLC, OLC, and OSC groups at different stages of the ovulatory cycle were analyzed by two-way ANOVA. These parameters as well as volumes of F1 follicles and intervals between ovulations were compared among the OILC hens (at 15 h after ovulation) and the YLC, OLC, and OSC hens (at 14.5 h after ovulation) using one-way ANOVA. Two-way ANOVA was also used to test effects of AG, LH and the AG by LH interaction on the total follicular volume and circulating concentrations of ovarian steroids in the YLC hens. Effects of AG on the total follicular volume and steroid concentrations in hens of the OILC group were examined by one-way ANOVA. Data on effects of E_2 _on the in vitro proliferative activity of granulosa and theca cells in the YLC and OSC hens were analyzed by two-way ANOVA for repeated measurements. The model used included the effect of the treatment (repeated factor), hen nested within the treatment (an error term), hen reproductive status and interaction between the status and treatment. Effects of testosterone and LH and their interaction on follicular cells of YLC hens were estimated by two-way ANOVA for repeated measurements (two factor repetition). Significant differences between means were determined using Tukey's test, which protect the significance tests of all combinations of pairs. A probability of *P *< 0.05 was considered to be statistically significant. Correlations between different reproductive parameters were estimated by Pearson's correlation coefficient (*r*), employing SigmaStat software package.

## Results

### Follicular growth and circulating steroid concentrations in hens of different ages and reproductive states

**Figure 1:**
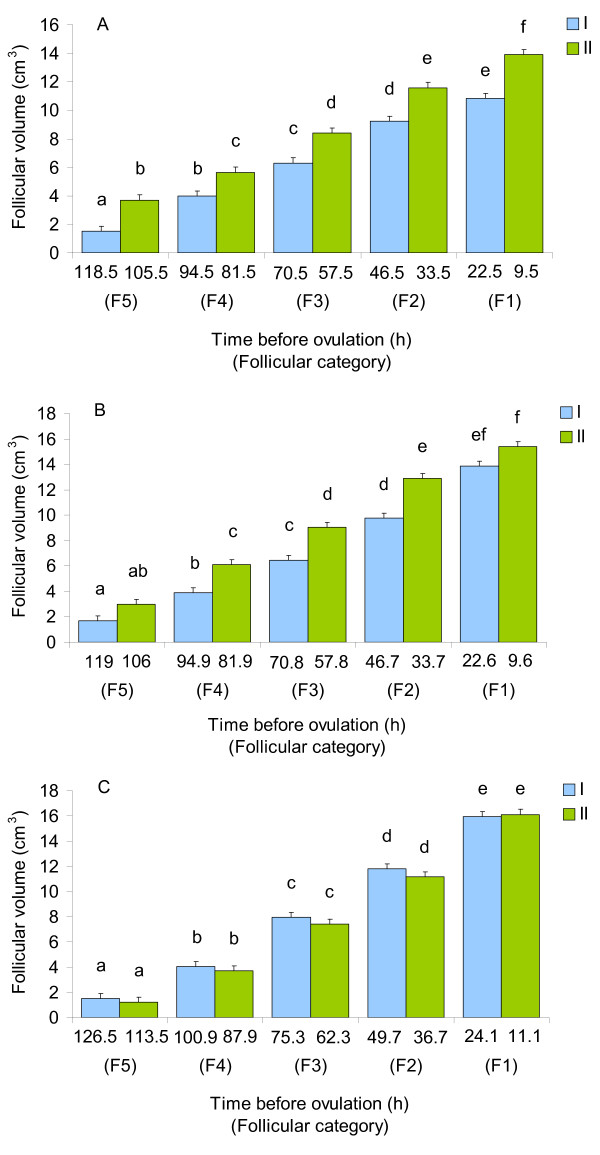
**Volumes of pre-ovulatory follicles with various degrees of maturation at different time points before ovulation in hens at distinct stages of the ovulatory cycle**. **A**) YLC hens: n = 6 (I and II). **B**) OLC hens: n = 6 (I and II). **C**) OSC hens: n = 10 (I) and n = 6 (II). I and II: stages of the hen ovulatory cycle (1.5 and 14.5 h after ovulation, respectively). Results are expressed as means ± SEM. Means with different letters differ significantly (at least *P *< 0.05).

The comparison of volumes of pre-ovulatory follicles of various categories at the distinct stages of the ovulatory cycle revealed that the growth dynamics of the follicular wall differed in hens of different reproductive statuses (Fig. 1). As indicated in the material and methods, the assessment of the growth of the follicular wall was based on the changes in the follicular volume. Chronological age by itself did not affect the follicular growth. Time before ovulation indicated in the figure demonstrates the passing of F5 follicle through five consecutive ovulatory cycles towards ovulation. In the YLC hens, the volume of the five pre-ovulatory follicles (from F5 to F1) rose between 1.5 and 14.5 h (at least *P *< 0.05) after ovulation followed by a transient termination of the growth (Fig. 1A). The pattern of the follicular growth in hens of the OLC group was very similar to that in the YLC hens, except that the volume of F1 and F5 follicles did not enlarge significantly in the middle of the ovulatory cycle (Fig. 1B). Whereas, there was no increase in the volume of all pre-ovulatory follicles in the OSC hens between 1.5 and 14.5 h after ovulation and, subsequently, the follicular growth was initiated (Fig. 1C).

No differences in the circulating levels of ovarian steroid hormones were found among hens from YLC, OLC, and OSC groups. Furthermore, in each group, the estradiol-17β level decreased between 1.5 and 14.5 h after ovulation (Table 2). The growth dynamics of all pre-ovulatory follicles during the ovulatory cycle was uniform within the groups of hens of the same age and reproductive status (Fig. 1). Thus, we combined volumes of F1-F5 follicles to examine a relationship between sex steroids, reproductive aging and the total growth pattern of the pre-ovulatory follicle wall. In both YLC and OLC hens, the total volume of pre-ovulatory follicles increased between 1.5 and 14.5 h after ovulation (*P *< 0.01). It negatively correlated with the plasma estradiol-17β level (Tables 2 and 3). By contrast, there was no growth of pre-ovulatory follicles in the middle of the cycle in OSC hens, with a positive correlation being present between the steroid concentration in plasma and total follicular volume. A positive correlation was also revealed between the plasma progesterone concentration and the time after ovulation in hens from YLC and OLC groups, whereas plasma P_4 _levels remained constant in hens of the OSC group (Tables 2 and 3). Moreover, the total volume of pre-ovulatory follicles at 1.5 h after ovulation was higher (*P *< 0.01) in the OSC group than in the YLC group. In OILC hens (the Control group, Experiment 2), the total follicular volume and plasma steroid concentrations at 15 h after ovulation were similar to those in OLC hens at 14.5 h after ovulation (Table 2). The volume of F1 follicles at this time of the cycle was the highest in hens of the OILC (Control) group (YLC: 13.9 ± 0.5 cm^3^; OLC: 15.4 ± 0.6 cm^3^; OSC: 16.1 ± 0.8 cm^3^; OILC: 17.0 ± 0.7 cm^3^, *P *< 0.05 vs. YLC). Furthermore, there was a positive correlation (*P *< 0.01) between the circulating levels of progesterone and the total follicular volume in OLC and OSC hens (Table 3). This positive correlation was also present when the groups (YLC, OLC, OSC, and OILC) were pooled (*P *< 0.001).

**Table 2:** The total volume of pre-ovulatory follicles and plasma steroid concentrations in hens of different ages and reproductive states

Group of hens	Interval between ovulations (h)	Stage/Estimated time after ovulation (h)	Number of hens	Total follicular volume (cm^**3**^)	Plasma estradiol-17β concentration (pg/ml)	Plasma testosterone concentration (pg/ml)	Plasma progesterone concentration (pg/ml)
YLC	24.0 ± 0.1^a^	I/1.5	6	31.8 ± 1.3^c^	486 ± 55	368 ± 66	224 ± 80
		II/14.5	6	43.2 ± 2.6**	248 ± 54**	372 ± 72	458 ± 120
OLC	24.1 ± 0.1^a^	I/1.5	6	35.7 ± 2.0	539 ± 23	510 ± 47	405 ± 84
		II/14.5	6	46.4 ± 2.5**	289 ± 29***	426 ± 51	773 ± 118
OSC	25.6 ± 0.2^b^	I/1.5	10	41.2 ± 1.9^d^	494 ± 50	410 ± 33	503 ± 129
		II/14.5	6	39.6 ± 1.6	335 ± 24*	385 ± 50	457 ± 96
OILC ^1^	24.4 ± 0.2^a^	II/15	7	46.8 ± 2.2	259 ± 32	365 ± 48	627 ± 136

^1 ^the Control group of the OILC hens (Experiment 2). Data were expressed as means ± SEM. Means with different superscripts within a column differ significantly (at least *P *< 0.01). Asterisks indicate differences between 1.5 and 14.5 h after ovulation within a group of hens: *P < 0.05; **P < 0.01; ***P < 0.001.

**Table 3:** Correlations between different reproductive parameters in hens of different ages and reproductive states

Reproductive parameters	r*	*P*
Plasma concentration of estradiol-17β vs. Total volume of pre-ovulatory follicles (F1-F5) in the YLC hens	- 0.62	< 0.05
Plasma concentration of estradiol-17β vs. Total volume of pre-ovulatory follicles (F1-F5) in the OLC hens	- 0.75	< 0.01
Plasma concentration of estradiol-17β vs. Total volume of pre-ovulatory follicles (F1-F5) in the OSC hens	0.60	< 0.05
Plasma concentration of progesterone in the YLC and OLC hens vs. Time after ovulation	0.53	< 0.01
Plasma concentration of progesterone vs. Total volume of pre-ovulatory follicles (F1-F5) in the OLC hens	0.78	< 0.01
Plasma concentration of progesterone vs. Total volume of pre-ovulatory follicles (F1-F5) in the OSC hens	0.62	< 0.01
Plasma concentration of progesterone vs. Total volume of pre-ovulatory follicles (F1-F5) in the YLC, OLC, OSC, and OILC hens	0.54	< 0.001

*Pearson's correlation coefficient.

### The impact of LH and aminoglutethimide (AG) injections on the growth of hen preovulatory follicles in relation to circulating ovarian steroids and reproductive aging

**Figure 2:**
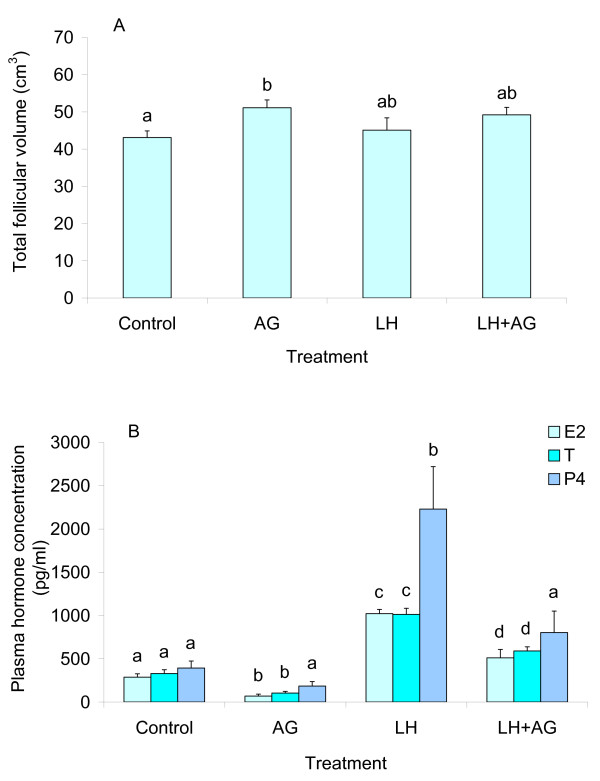
**Reproductive parameters in YLC hens injected with LH and/or AG**. The hens were subcutaneously injected with saline or 100 mg AG at 9.5 and 11.5 h after ovulation. Immediately after the second treatment with AG, the hens were intravenously injected with saline or 100 μg oLH. The hens were sacrificed at 3.5 h after the treatment with AG or oLH, corresponding to 15 h after ovulation. The schedule of the treatments is given in Table 1. Numbers of hens: n = 9 (Control and AG), n = 7 (LH) and n = 8 (LH+AG). Results are expressed as means ± SEM. **A**) The total volume of pre-ovulatory follicles (F1-F5). Means with different letters differ significantly (at least *P *< 0.05). **B**) Plasma concentrations of estradiol-17β (E_2_), testosterone (T) and progesterone (P_4_). Means for each hormone with different letters differ significantly (at least *P *< 0.05).

Applications of AG to YLC hens caused a rise in the total volume of pre-ovulatory follicles and a decline in levels of circulating steroids (Fig. 2A and 2B). The decline in progesterone was, however, not significant. In addition, the gain in the total follicular volume induced by AG was associated with a decrease in the plasma estradiol-17β concentration (*r *= -0.54, *P *< 0.05). Treatments with LH caused a rise in the concentrations of the steroid hormones, and AG partly attenuated the stimulatory effect of LH (Fig. 2B). Although LH did not affect the follicular growth in young hens (Fig. 2A), there was a negative correlation between the plasma level of testosterone and the total follicular volume in hens of the LH-treated group (*r *= -0.79, *P *< 0.05). Furthermore, a positive correlation was revealed between the follicular volume and the plasma estradiol-17β concentration in hens injected with LH+AG (*r *= 0.77, *P *< 0.05).

In contrast to YLC hens, there was no influence of AG on the total volume of preovulatory follicles and the concentration of circulating testosterone in OILC hens (Table 4). Furthermore, the effect of AG on the plasma progesterone level was more profound in the OILC group than in the YLC group, resulting in a four-fold decline in P_4 _values in this group (*P *< 0.01).

**Table 4:** The total volume of pre-ovulatory follicles and plasma ovarian steroid concentrations in the OILC hens injected with AG

Treatment group	Number of hens	Total follicular volume (cm^**3**^)	Plasma estradiol-17β concentration (pg/ml)	Plasma testosterone concentration (pg/ml)	Plasma progesterone concentration (pg/ml)
Control	7	46.8 ± 2.2	259 ± 32^a^	365 ± 48	627 ± 136^a^
AG	7	46.3 ± 3.0	≤ 50^b^	344 ± 62	142 ± 55^b^

Data were expressed as means ± SEM. Means with different superscripts within a column differ significantly (at least *P *< 0.01).

### Proliferative responses of follicular cells to hormones in vitro

**Figure 3:**
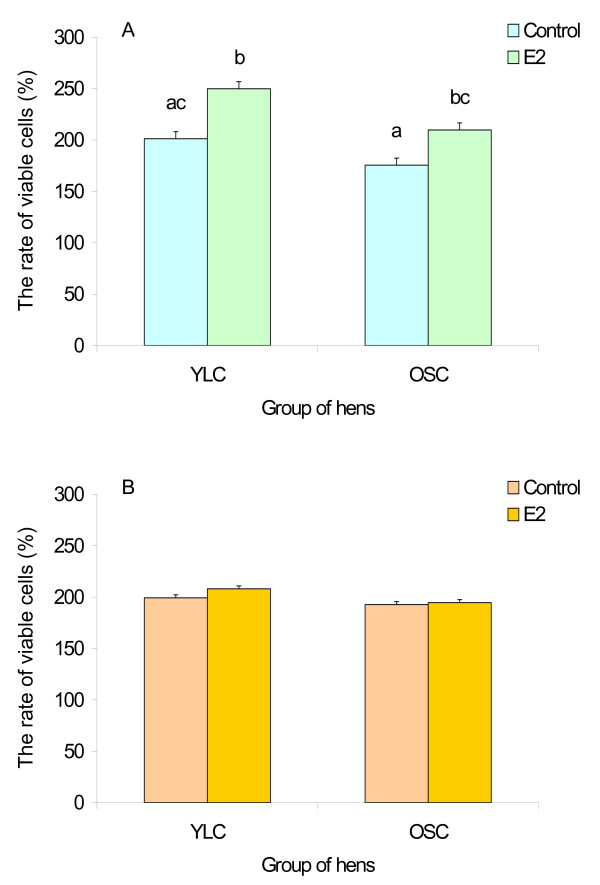
**Effects of estradiol-17β on proliferation of granulosa (A) and theca cells (B) from F3 follicles**. Each bar represents the relative number of viable cells after 40 h of culture expressed in percent of the number of the cells prior to culture. Results are expressed as means ± SEM. Means with different letters differ significantly (at least *P *< 0.01).

**Figure 4:**
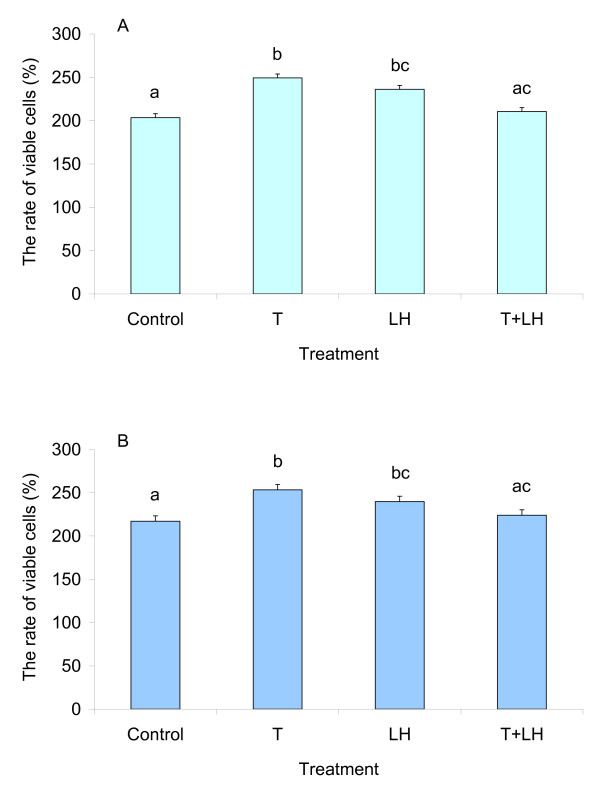
**Effects of testosterone and LH on proliferation of granulosa (A) and theca cells (B) from F3 follicles of young hens**. Each bar represents the relative number of viable cells after 40 h of culture expressed in percent of the number of the cells prior to culture. Results are expressed as means ± SEM. Means with different letters differ significantly (at least *P *< 0.05).

Direct effects of estradiol-17β, testosterone and LH on the proliferative activity of granulosa and theca cells were examined in vitro using F3 pre-ovulatory follicles. After 40 h of culture in the control medium, the number of viable cells was approximately doubled in all cases (Fig. 3 and 4). The addition of 1 ng/ml E_2 _to the culture medium caused an acceleration of the proliferation of granulosa cells in both YLC and OSC groups (at least *P *< 0.01, Fig. 3A). There was no considerable difference in the response to the E_2_-challenge between the two groups. In contrast, estradiol-17β did not exert any effect on the proliferative activity of theca cells (Fig. 3B).

We found a relationship between the total follicular volume and the plasma level of testosterone in YLC hens treated with LH. Therefore, young birds were used for testing effects of these hormones on the cell proliferation in vitro. When added separately to the culture medium, testosterone and LH increased the relative number of viable granulosa cells as compared to that in Controls (*P *< 0.01, Fig. 4A). However, this stimulatory effect disappeared when the cells were treated with a combination of these two hormones. The same phenomenon was also observed in the theca cells (Fig 4B). Two-way repeated-measures ANOVA revealed that the proliferative activities of both granulosa and theca cells of YLC hens were depended on the interaction between testosterone and LH (at least *P *< 0.01).

## Discussion

A series of new findings, elucidating relationships between reproductive aging, sex steroids and the growth pattern of the pre-ovulatory follicle wall, were obtained in the present research. The data indicate for the first time that the tissue growth of hen pre- ovulatory follicles may be differently affected by the cyclic alterations in the production of ovarian steroid hormones in young and reproductive aged birds. In the present study we did not consider steroid receptors. But, changes in the expression of the steroid receptors may also influence the growth pattern of follicles.

The wall of pre-ovulatory follicles in YLC hens grows between 1.5 and 14.5 h after ovulation followed by a transient termination of growth. This pattern is similar to that we published previously for the theca layer in young hens [17]. The increase in the size of pre-ovulatory follicles in the middle of the ovulatory cycle was accompanied by the decrease in the plasma estradiol-17β concentration, which had also been observed by other authors [28,29]. The theca layer is known to produce less estradiol-17β as pre-ovulatory follicles pass from F5 to F1 category [30,31]. This phenomenon is due to the follicular differentiation rather than the follicular growth itself [26]. We have shown previously that the theca growth occurs in the middle of the ovulatory cycle (between 9 and 16 h after ovulation), whereas its differentiation occurs after the growth termination induced by the pre-ovulatory hormone surge [17]. On the other hand, AG applications resulted in an increase in the follicular volume in YLC hens. This increase was negatively correlated with the estradiol-17β concentrations. Taken together these data provide reason to assume that a decline in the production of estradiol-17β in the middle of the hen ovulatory cycle may be one of the stimuli causing the follicular tissues to grow. However, this relationship could not be due to a direct suppressive action of estradiol-17β on proliferation of follicular cells, which was observed in mammals [32]. In the present study, E_2 _raised the proliferative activity of granulosa cells and did not affect that of theca cells. Both of these cell types express estrogen receptors [33,34]. Therefore, the inverse association of circulating E_2 _with the follicular size in young hens appears to reflect its indirect inhibitory effect on the growth of the follicular wall. This inhibitory effect may be due to a regulatory action of estradiol-17β on insulin-like growth factor (IGF) system inherent in avian ovarian follicles [35,36].

Our previous data [17] have suggested that the pre-ovulatory rise in ovarian steroid hormones and/or LH is involved in the short-time termination of the thecal growth in young hens. Tischkau et al. [37] revealed a reduction in the granulosa proliferative activity after the pre-ovulatory LH surge. In the present study, the total follicular volume in LH-treated YLC hens negatively correlated with the plasma testosterone concentration, suggesting an implication of testosterone in suppression of the growth of the follicular tissues. It is unlikely that at basal concentrations testosterone can affect proliferation of follicular cells in young hens, since the steroid was not related to the follicle size in the control and AG-treated birds. Further, in vitro experiments showed that testosterone and LH at high concentrations exert stimulatory effects on the proliferative activity of granulosa and theca cells. This effect, however, was suppressed when the cells were treated with a combination of both hormones. Therefore, it seems plausible that the interaction of testosterone and LH on the tissue growth could be responsible for the unchanged total follicular volume in the LH-treated hens. These data provide reason to assume that the short-time termination of the growth of the follicular wall during the pre-ovulatory hormone surge in young hens is determined, at least in part, by crosstalk between testosterone and LH.

The growth pattern of the pre-ovulatory follicle wall during the ovulatory cycle in OSC hens is the opposite of that in YLC hens. Thus, the pre-ovulatory surge of the hormones obviously does not suppress the follicular growth in OSC hens. In accordance to previous works [15,16], we have not found differences in the circulating levels of ovarian steroid hormones between birds of different ages and reproductive statuses. Further investigations are required to establish the functional details of the crosstalk between testosterone and LH in old hens.

Although the estradiol-17β concentration declines in the middle of the ovulatory cycle in OSC hens, the correlation between the concentration and total follicular volume is opposite to that in hens with high laying performance. This positive relationship is consistent with the direct stimulatory effect of estradiol-17β on the proliferative activity of the hen granulosa cells observed in vitro. Taken together these data suggest that mechanisms, determining the indirect inhibitory action of E_2 _on the follicular growth in YLC hens, are impaired in OSC hens.

In reproductive aged mammals, the availability of anovulatory cycles/non-ovulatory follicles is a frequent occurring phenomenon [2,10]. The enhanced frequency of missing eggs/anovulatory cycles within clutch is one of the reasons of impaired reproductive function in old hens as well [13]. Progesterone and presumably testosterone are involved in the induction of ovulation in laying hens [38,39]. In the present study, we did not observe considerable differences in the total follicular volume and plasma steroid concentrations between OILC hens and hens of other groups. However, the response to AG treatments was not similar in YLC and OILC groups. In contrast to the young hens, AG did not affect the follicular growth and the plasma concentration of testosterone in OILC hens. Hence, the absence of changes in the tissue growth of pre-ovulatory follicles in response to AG is due to the failure of the latter to reduce the circulating level of testosterone. Conversely, the fall in the level of progesterone in OILC hens was two times greater than in YLC hens, whereas the decrease in the level of estradiol-17β was closely similar in both groups. This suggests that a part of progesterone could be converted to testosterone. It has been shown that AG interrupts cholesterol side-chain cleavage by the cytochrome P-450 [19] and P_4 _synthesis takes place up-stream from the site of T synthesis. The largest hen pre-ovulatory follicle (F1) produces most of progesterone, whereas the secretion of androgens by this follicle abruptly decreases [38]. F1 follicle loses the ability to convert progesterone to androstenedione by 12 h before ovulation, contributing to the pre-ovulatory rise in P_4_[31]. Therefore, it may be suggested that the F1 follicle of OILC hens retains, at least in part, this ability, which can result in a lower pre-ovulatory gain in progesterone [40] and the high incidence of anovulatory cycles. It should be noted that the F1 follicle secretes a negligible quantity of estradiol-17β and, consequently, the conversion of testosterone to estradiol-17β by this follicle could not contribute considerably into the circulating level of estradiol-17β in OILC hens treated with AG.

## Conclusions

The present study demonstrates for the first time that the growth pattern of pre-ovulatory follicle wall during the ovulatory cycle gradually changes in the course of reproductive aging in laying hens. Estradiol-17β plays a dual role in this adjustment; it can stimulate the follicular growth in old hens with short clutch, whereas its inverse association with the follicular size appears to reflect its indirect inhibitory action on the tissue growth of pre-ovulatory follicles in young birds. The novel findings also suggest an involvement of testosterone and LH in the transitory termination of the follicular growth during the pre-ovulatory hormone surge in young hens. Furthermore, the persisting ability of the largest pre-ovulatory follicle to convert progesterone into androgens may be responsible for the anovulatory cycles in old hens.

## Competing interests

The authors declare that they have no competing interests.

## Authors' contributions

IYL designed the study, performed the experiments and prepared manuscript. VAL participated in all experiments and discussion of the study design and results. RG conceived the idea of the study and participated in its design, coordination, and discussion of results. NP directed all aspects of the studies and was responsible for the critical revision of the manuscript. All authors (except VAL) read and approved the final manuscript.

## References

[B1] Macklon NS Fauser BC Aspects of ovarian follicle development throughout life Horm Res 1999 52 161 170 10.1159/000023456 10725781

[B2] Peluso JJ Steger RW Huang H Meites J Pattern of follicular growth and steroidogenesis in the ovary of aging cycling rats Exp Aging Res 1979 5 319 333 10.1080/03610737908257208 574826

[B3] Lerner SP Meredith S Thayne WV Butcher RL Age-related alterations in follicular development and hormonal profiles in rats with 4-day estrous cycles Biol Reprod 1990 42 633 638 10.1095/biolreprod42.4.633 2112028

[B4] Gougeon A Ovarian follicular growth in humans: ovarian aging and population of growing follicles Maturitas 1998 30 137 142 10.1016/S0378-5122(98)00069-3 9871908

[B5] Van Zonneveld P Scheffer GJ Broekmans FJ Blankenstein MA de Jong FH Looman CW Habbema JD de Velde ER Do cycle disturbances explain the age-related decline of female fertility? Cycle characteristics of women aged over 40 years compared with a reference population of young women Hum Reprod 2003 18 495 501 10.1093/humrep/deg138 12615813

[B6] Chakraborty TR Gore AC Aging-related changes in ovarian hormones, their receptors, and neuroendocrine function Exp Biol Med (Maywood) 2004 229 977 987 15522833 10.1177/153537020422901001

[B7] Nelson JF Bergman MD Karelus K Felicio LS Aging of the hypothalamo-pituitary-ovarian axis: hormonal influences and cellular mechanisms J Steroid Biochem 1987 27 699 705 10.1016/0022-4731(87)90139-7 3320554

[B8] Burger HG Dudley E Mamers P Robertson D Groome N Dennerstein L The ageing female reproductive axis I Novartis Found Symp 2002 242 161 167 full_text 11855686

[B9] Klein NA Battaglia DE Miller PB Branigan EF Giudice LC Soules MR Ovarian follicular development and the follicular fluid hormones and growth factors in normal women of advanced reproductive age J Clin Endocr Metab 1996 81 1946 1951 10.1210/jc.81.5.1946 8626862

[B10] Santoro N Isaac B Neal-Perry G Adel T Weingart L Nussbaum A Thakur S Jinnai H Khosla N Barad D Impaired folliculogenesis and ovulation in older reproductive aged women J Clin Endocr Metab 2003 88 5502 5509 10.1210/jc.2002-021839 14602797

[B11] Peluso JJ Downey C Pattern of follicular development during the estrous cycle of aged rats Cell Tissue Res 1982 225 229 234 10.1007/BF00216232 6889465

[B12] Williams JB Sharp PJ Ovarian morphology and rates of ovarian follicular development in laying broiler breeders and commercial egg-producing hens Br Poult Sci 1978 19 387 395 10.1080/00071667808416490

[B13] Lillpers K Wilhelmson M Age-dependent changes in oviposition pattern and egg production traits in the domestic hen Poult Sci 1993 72 2005 2011 8265489 10.3382/ps.0722005

[B14] Zakaria AH Miyaki T Imai K The effect of aging on the ovarian follicular growth in laying hens Poult Sci 1983 62 670 674 6866903 10.3382/ps.0620670

[B15] Johnson PA Dickerman RW Bahr JM Decreased granulosa cell luteinizing hormone sensitivity and altered thecal estradiol concentration in the aged hen, Gallus domesticus Biol Reprod 1986 35 641 646 10.1095/biolreprod35.3.641 3790665

[B16] Joyner CJ Peddie MJ Taylor TG The effect of age on egg production in the domestic hen Gen Comp Endocrinol 1987 65 331 336 10.1016/0016-6480(87)90117-1 3557097

[B17] Lebedev VA Lebedeva IY Grossmann R Kuzmina TI Parvizi N Ovulatory cycle-related alterations in the thecal growth and membrane protein content of theca layers of hen preovulatory follicles Theriogenology 2006 66 217 223 10.1016/j.theriogenology.2005.11.004 16325901

[B18] Warren DC Scott HM The time factor in egg formation Poult Sci 1935 14 195 207

[B19] Uzgiris VI Graves P Salhanick HA Ligand modification of corpus luteum mitochondrial cytochrome P-450 spectra and cholesterol mono oxygenation: an assay of enzyme-specific inhibitors Biochemistry 1977 16 593 600 10.1021/bi00623a006 836802

[B20] Johnson AL van Tienhoven A Effects of aminoglutethimide on luteinizing hormone and steroid secretion, and ovulation in the hen, Gallus Domesticus Endocrinology 1984 114 2276 2283 10.1210/endo-114-6-2276 6373241

[B21] Etches RJ MacGregor HE Morris TF Williams JB Follicular growth and maturation in the domestic hen (Gallus domesticus) J Reprod Fertil 1983 67 351 358 6339717 10.1530/jrf.0.0670351

[B22] Gilbert AB Evans AJ Perry MM Davidson MH A method for separating the granulosa cells, the basal lamina and the theca of the preovulatory ovarian follicle of the domestic fowl (Gallus domesticus) J Reprod Fertil 1977 50 179 181 864645 10.1530/jrf.0.0500179

[B23] Tilly JL Johnson AL Regulation of androstenedione production by adenosine 3',5'-monophosphate and phorbol myristate acetate in ovarian thecal cells of the domestic hen Endocrinology 1989 125 1691 1699 10.1210/endo-125-3-1691 2474440

[B24] Roberts RD Sharp PJ Burt DW Goddard C Insulin-like growth factor-I in the ovary of the laying hen: gene expression and biological actions on granulosa and thecal cells Gen Comp Endocrinol 1994 93 327 336 10.1006/gcen.1994.1037 8194735

[B25] Yao HHC Bahr JM Chicken granulosa cells show differential expression of epidermal growth factor (EGF) and luteinizing hormone (LH) receptor messenger RNA and differential responsiveness to EGF and LH dependent upon location of granulosa cells to the germinal disc Biol Reprod 2001 64 1790 1796 10.1095/biolreprod64.6.1790 11369610

[B26] Hernandez-Vertiz A Gonzalez del Pliego M Velazquez P Pedernera E Morphological changes in the theca layer during the maturation of the preovulatory ovarian follicle of the domestic fowl Gen Comp Endocrinol 1993 92 80 87 10.1006/gcen.1993.1145 8262359

[B27] Sirotkin AV Grossmann R Role of tyrosine kinase- and MAP kinase-dependent intracellular mechanisms in control of ovarian functions in the domestic fowl (Gallus domesticus) and in mediating effects of IGF-II J Reprod Develop 2003 49 99 106 *Erratum ***49(4)** 10.1262/jrd.49.99 14967954

[B28] Shodono M Nakamura T Tanabe Y Wakabayashi K Simultaneous determinations of oestradiol-17 beta, progesterone and luteinizing hormone in the plasma during the ovulatory cycle of the hen Acta Endocrinol (Copenh) 1975 78 565 573 1173019 10.1530/acta.0.0780565

[B29] Yamamura N Takeishi M Goto H Tagami M Mizutani T Miyamoto K Doi O Kamiyoshi M Expression of messenger RNA for gonadotropin receptor in the granulosa layer during the ovulatory cycle of hens Comp Biochem Phys A 2001 129 327 337 10.1016/s1095-6433(00)00350-0 11423305

[B30] Bahr JM Wang SC Huang MY Calvo FO Steroid concentrations in isolated theca and granulosa layers of preovulatory follicles during the ovulatory cycle of the domestic hen Biol Reprod 1983 29 326 334 10.1095/biolreprod29.2.326 6640023

[B31] Robinson FE Etches RJ Ovarian steroidogenesis during follicular maturation in the domestic fowl (Gallus domesticus) Biol Reprod 1986 35 1096 1105 10.1095/biolreprod35.5.1096 2950935

[B32] Ranson EJ Picton HM Hunter MG Effects of testosterone and oestradiol on [^3^H]-thymidine incorporation by porcine granulosa and theca cells Anim Reprod Sci 1997 47 229 236 10.1016/S0378-4320(97)00004-3 9329864

[B33] Kamiyoshi M Niwa T Tanaka K Nuclear estrogen receptor bindings in granulosa cells and estradiol-17 beta contents in follicular membranes of the ovary of the hen during the ovulatory cycle Gen Comp Endocrinol 1986 61 428 435 10.1016/0016-6480(86)90229-7 3956994

[B34] Hrabia A Wilk M Rzasa J Expression of alpha and beta estrogen receptors in the chicken ovary Folia Biol (Krakow) 2008 56 187 191 10.3409/fb.56_3-4.187-191 19055045

[B35] Onagbesan OM Vleugels B Buys N Bruggeman V Safi M Decuypere E Insulin-like growth factors in the regulation of avian ovarian functions Domest Anim Endocrin 1999 17 299 313 10.1016/S0739-7240(99)00046-6 10527132

[B36] Radecki SV Capdevielle MC Buonomo FC Scanes CG Ontogeny of insulin-like growth factors (IGF-I and IGF-II) and IGF-binding proteins in the chicken following hatching Gen Comp Endocrinol 1997 107 109 117 10.1006/gcen.1997.6898 9208310

[B37] Tischkau SA Neitzel LR Walsh JA Bahr JM Characterization of the growth center of the avian preovulatory follicle Biol Reprod 1997 56 469 474 10.1095/biolreprod56.2.469 9116148

[B38] Etches RJ The ovulatory cycle of the hen Crit Rev Poult Biol 1990 2 293 318

[B39] Rangel PL Sharp PJ Gutierrez CG Testosterone antagonist (flutamide) blocks ovulation and preovulatory surges of progesterone, luteinizing hormone and oestradiol in laying hens Reproduction 2006 131 1109 1114 10.1530/rep.1.01067 16735550

[B40] Holmes DJ Thomson SL Wu J Ottinger MA Reproductive aging in female birds Exp Gerontol 2003 38 751 756 10.1016/S0531-5565(03)00103-7 12855282

